# Impact of steroid withdrawal on subclinical graft injury after liver transplantation: A propensity score-matched cohort analysis

**DOI:** 10.3389/frtra.2023.1124551

**Published:** 2023-02-22

**Authors:** Alejandro Campos-Murguia, Emily A. Bosselmann, Björn Hartleben, Heiner Wedemeyer, Bastian Engel, Richard Taubert, Elmar Jaeckel

**Affiliations:** ^1^ Department of Gastroenterology, Hepatology, Infectious Diseases and Endocrinology, Hannover Medical School, Hannover, Germany; ^2^ Institute for Pathology, Hannover Medical School, Hannover, Germany

**Keywords:** rejection, immunosuppression, individualized therapies, personalized medicine, fibrosis

## Abstract

Subclinical graft injuries in orthotopic liver transplantation may threaten long-term graft survival and could be the result of chronic under-immunosuppression. It is not known whether steroid withdrawal increases the risk of subclinical immune responses against the graft. This retrospective single-center study aimed to assess the risk of subclinical graft damage after steroid withdrawal within the first nine months after orthotopic liver transplantation in the first three years after transplantation in a prospective cohort of surveillance biopsies using a propensity score matching analysis. Of 355 patients, 109 patients underwent surveillance biopsies between eleven and 36 months after liver transplantation. Thirty-seven patients discontinue steroids within the first nine months and 72 later than nine months after transplantation. The matching led to 28 patients per group. Patients with autoimmune hepatitis, primary biliary cholangitis, and hepatocarcinoma were excluded by the propensity score matching unintentionally. Patients who discontinued steroids had a trend toward lower levels of immunosuppression at the time of surveillance biopsy. Steroid withdrawal in the first nine months was not associated with an increased risk of subclinical T cell-mediated rejection, graft inflammation, or liver graft fibrosis in the matched cohort with patients with a low frequency of autoimmune liver diseases. There were also no differences in the development of metabolic diseases. In conclusion, steroid withdrawal within the first nine months after transplantation, as assessed by surveillance biopsies, does not increase the risk of subclinical graft injuries or fibrosis at least in liver transplant recipient without or a low prevalence of autoimmune liver diseases.

## 1. Introduction

The liver is immunologically privileged compared to other solid organ transplants. This is exemplified by the low prognostic relevance of early T cell-mediated rejection (TCMR) episodes, the low incidence of chronic as well as antibody-mediated rejection (ABMR), and the highest rates of spontaneous operational tolerance after orthotopic liver transplantation (OLT) (1, 2). Consequently, the strength of the immunosuppression (IS) regimen after OLT is the lowest of all solid organ transplants.

Steroids have been one of the cornerstones of IS protocols since the early days of OLT (3). Due to their poor long-term safety profile, which includes an increased risk of steroid-related hypertension (HTN), new onset of diabetes after transplant (NODAT), and osteoporosis (4), and the availability of other immunosuppressive drugs such as calcineurin inhibitors (CNI), the use of steroids has been relegated to the first months after OLT. There is, however, no consensus regarding the optimal and safer moment for their withdrawal. Steroid suspension protocols differ widely between transplantation centers, ranging from steroid-free protocols to indefinite therapies. Previous randomized controlled trials have evaluated free or short-term steroid protocols with the development of biopsy proven acute rejection as their primary outcome (5–8). Biopsy proven acute rejection and graft loss are just presenting the tip of an iceberg of immune responses against the graft, subclinical TCMR (subTCMR) might lead to graft fibrosis even in the absence of clinically seen acute rejections (9). In addition, the published studies on steroid withdrawal were dominated by hepatitis C virus (HCV) reinfections, which are clinically less relevant nowadays, ciclosporin A (CyA) based IS regimens, which have largely been superseded by tacrolimus (TAC) or did not include surveillance biopsies (svLbx) (5–8).

SvLbx exhibited graft injury patterns in the majority of biopsies although liver enzymes were normal or only marginally elevated (10–12). Some studies report an association of subclinical graft injury with slowly progressive liver fibrosis and the upregulation of rejection associated transcripts (9, 10, 12, 13). Furthermore, patients with relevant subclinical graft injury including subTCMR are probably not candidates for further IS reduction (11, 14). So far, the only method available to identify subclinical allograft damage is through svLbx. However, few transplantation centers have implemented these protocols routinely in their programs, further limiting the availability of evidence on risk factors, prognosis, and treatment of these lesions. Nonetheless, the latest Banff consensus document outlines thresholds for subclinical graft injury for IS reduction (15). To our knowledge, there is no evidence as to whether there is an increased risk of subclinical graft injuries after steroid withdrawal in OLT.

The aim of this retrospective single center study was to evaluate whether steroid discontinuation within the first months after OLT increased the risk of subclinical graft injuries on svLbx within the first three years after OLT.

## 2. Material and methods

### 2.1. Study outline

This is a single-center retrospective study on the safety of steroid withdrawal assessed by svLbx after OLT. Since most centers withdraw steroids around six months after OLT, we evaluated a cut-off of nine months, considering a tapering period of three months to complete withdrawal. Once the groups were established, a propensity score matched (PSM) analysis was performed to balance the variables and reduce the risk of bias.

### 2.2. Subjects

The Hannover Medical School has been conducting a program of svLbx after OLT since 2008. We included all liver recipients (LR) without replicative viral hepatitis (HCV-RNA or HBs-Ag negativity) who had one representative liver biopsy between eleven and 36 months after OLT and agreed to participate in our prospective svLbx program. If a patient had two svLbx during this period, only the first was included. Participation in the svLbx program was voluntary and offered to all liver transplanted patients without contraindications for a biopsy.

Since 2017 the standard IS regimen in the first year suggests a steroid withdrawal after the first four months following OLT. Steroids are usually withdrawn stepwise over up to four months to reduce the risk of adrenal insufficiency. Before 2017 steroids were withdrawn on an individual basis. Since we screened patients who had a svLbx since 2008, a considerable proportion of patients had CyA as their primary IS, which is not the up to date IS standard, usually based in TAC. Therefore, we excluded these patients to have a more representative cohort of the current management.

This study was approved by the local Ethics Committee (protocol number 933 for project Z2 of comprehensive research center 738). Written informed consent was obtained from all subjects in advance.

### 2.3. Histologic evaluation

All biopsies were blindly reviewed by experienced liver pathologists trained on the most recent BANFF guidelines (15), and classified according to the following up-to-date scores: Rejection Activity Index (RAI) (15), Ishak modified Histological Activity Index (HAI) (16). Liver Allograft Fibrosis score (LAF) (17), Ishak fibrosis score (16), and BanffMini score (portal tract inflammation ≤1, interface hepatitis ≤1, central perivenulitis ≤1, lobular inflammation = 0, biliary inflammation = 0, endothelialitis = 0, portal microvasculitis = 0 and periportal fibrosis ≤3) (15).

### 2.4. Definitions

Discontinuation of steroids at or within the first nine months after OLT was referred to as “early steroid withdrawal” (ESW) and discontinuation of steroids after nine months of OLT was referred to as “late steroid withdrawal” (LSW).

SubTCMR was defined by a Banff RAI ≥ 1 + 1 + 1 (portal, bile duct, venous endothelial inflammation) in the absence of significant liver enzyme elevations (AST, ALT, AP ≤ 2 × ULN). Indeterminate TCMR was defined as RAI = 2 and clinical TCMR as RAI ≥ 1 + 1 + 1 plus relevant liver enzyme elevations (>2x ULN).

Regarding the assessment of the IS level, we used a semi-quantitative score, a modification of a previously reported dosage-based score (18), considering level aims instead of the drug dosages. Assigning two points to a TAC aim of >5 ng/ml and a CyA aim of >60 ng/ml, one point to a TAC aim of 3–5 ng/ml, and a CyA aim of 30–60 ng/ml and finally 0.5 points to a TAC aim of <3 ng/ml and a CyA aim of <30 ng/ml as a substitute for the CNI dosages (11).

The diagnoses of NODAT and new-onset HTN were made in those patients who did not have these diagnoses before the OLT and that had them at the time of the svLbx according to the criteria of the treating physician. Lastly, the biliary complications were defined by the presence of biliary stenosis or bile leakage requiring an intervention for their resolution before the svLbx.

### 2.5. Statistical analysis

PSM was used to address bias and balance the variables that could influence the probability of treatment assignment, in this case, steroid withdrawal at or before nine months after OLT. Covariates chosen were those that could influence the probability of steroid discontinuation and that exhibit a statistical difference in the bivariate analysis before the matching. The propensity score was estimated using logistic regression. The matching procedure was performed using the nearest available neighbor, without replacement in a 1:1 ratio and a caliper of 0.2 (19, 20). Standardized mean difference (SMD) lower than 0.1 and a variance ratio below 2, were considered signs of balance (21).

The distribution of the variables was evaluated with the Shapiro–Wilk test and Quantile-Quantile plots. Since most numerical variables showed a non-normal distribution, all variables are presented in median and IQR, while categorical variables are presented in frequencies and percentages. For comparisons between groups, Mann–Whitney *U* for numerical variables, and the *χ*^2^ test for categorical variables were performed. A *p*-value of <0.05 was considered statistically significant.

All statistical analysis was done using R version 4.1.2, with the MatchIt package for the PSM analysis (22).

## 3. Results

### 3.1. Patients

**Figure 1:**
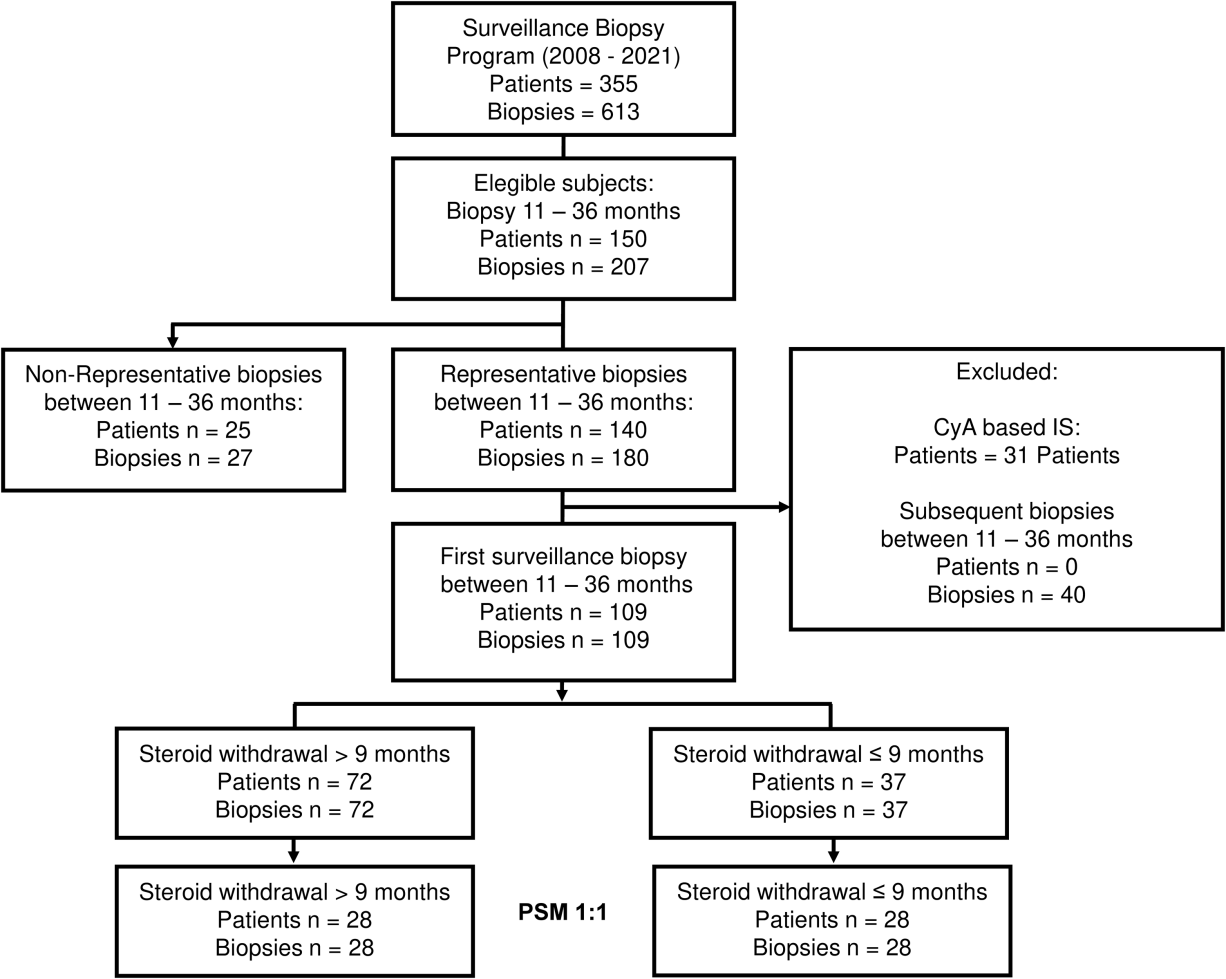
Flowchart of the patient selection. Flowchart outlining the patient's selection process and the number of subjects selected after the PSM. IS, immunosuppression; PSM, propensity score matched, CyA, cyclosporine.

In total, 355 patients with 613 biopsies have been screened in the surveillance biopsy program since 2008 (Figure 1). Of these, 150 patients had 207 surveillance biopsies between eleven and 36 months after OLT. We excluded non-representative biopsies and patients with CyA as their primary IS, and for those patients with more than one biopsy in this period, only the first svLbx was included, to avoid a bias of IS adjustment based on this first svLbx as regularly performed in our personalized IS program (11). Hence, 109 patients with 109 biopsies were included in the first analysis. Of these, 72 (66.1%) patients were in the LSW group, who continued steroids beyond nine months after LT, and 37 (33.9%) in the ESW group, who discontinued steroids within the first nine months after LT (Figure 1). Fifty-five (76.4%) patients in the LSW group had steroids at the time of the biopsy. Patients with ESW were significantly older [median age, 54.0 (28.0, 67.0) years] than those with LSW [median age, 50.0 (17.0, 65.0) years], had a lower incidence of rejection within the first nine months after OLT (1 (2.7%) compared to 19 (26.4%); *p* = 0.01), and had a trend toward significance difference in the etiology of the liver disease (*p* = 0.06), primarily due to a lower prevalence of autoimmune hepatitis (AIH) [0 compared to 8 (11.1%)] and primary sclerosing cholangitis (PSC) (2 (5.4%) compared to 13 (18.1%)). A statistical difference was also found regarding the time from the OLT to the surveillance biopsy, 17.0 (12.8, 25.0) months in the LSW group vs. 13.0 (12.0, 17.0) months in the ESW group, however, this difference was not considered clinically relevant (Table 1).

**Table 1:** Baseline characteristics of the patients before and after PSM analysis.

	Before PSM			PSM		
	LSW after OLT (*n* = 72)	ESW after OLT (*n* = 37)	*p*-value	LSW after OLT (*n* = 28)	ESW after OLT (*n* = 28)	*p*-value
Male sex	46 (63.9)	20 (54.1)	0.43	20 (71.4)	16 (57.1)	0.40
Age at OLT (years)	50.0 (17.0, 65.0)	54 (28.0, 67.0)	0.01	52.0 (47.0, 58.2)	54.0 (48.5, 58.3)	0.71
Etiology			0.06			0.99
AIH	8 (11.1)	0		0	0	
Alcohol	9 (12.5)	5 (13.5)		3 (10.7)	5 (17.9)	
Cryptogenic	8 (11.1)	6 (16.2)		7 (25.0)	5 (17.9)	
Polycystic liver disease	8 (11.1)	3 (8.1)		2 (7.1)	3 (10.7)	
HBV	6 (8.3)	7 (18.9)		5 (17.9)	6 (21.4)	
HCC	0	1 (2.7)		0	0	
HCV	6 (8.3)	5 (13.5)		5 (17.9)	3 (10.7)	
Metabolic diseases	7 (9.7)	1 (2.7)		1 (3.6)	1 (3.6)	
NAFLD/NASH	2 (2.8)	3 (8.1)		2 (7.1)	2 (7.1)	
Other	4 (5.6)	1 (2.7)		1 (3.6)	1 (3.6)	
PBC	1 (1.4)	3 (8.1)		0	0	
PSC	13 (18.1)	2 (5.4)		2 (7.1)	2 (7.1)	
BMI at OLT	23.7 (14.6, 45.0)	22.3 (18.3, 31.9)	0.50	24.0 (21.7, 26.4)	23.8 (20.4, 26.6)	0.67
DM pre-OLT	9 (12.5)	3 (8.1)	0.71	7 (25.0)	2 (7.1)	0.15
HTN pre-OLT	17 (23.6)	13 (35.1)	0.29	6 (21.4)	11 (39.3)	0.25
Graft rejection ≤9 months	19 (26.4)	1 (2.7)	0.01	0	0	–
Months from OLT to surveillance biopsy	17 (12.8, 25.0)	13 (12.0, 17.0)	0.01	15 (12.0, 24.3)	13 (12.0, 17.3)	0.42

Data is provided as no. (%) or median [IQR].

AIH, autoimmune hepatitis; BMI, body max index; DM, diabetes mellitus; ESW, early steroid withdrawal; HBV, hepatitis B virus; HCC, hepatocellular carcinoma; HCV, hepatitis C virus; HTN, hypertension; LSW, late steroid withdrawal; NAFLD, non-alcoholic liver disease; NASH, non-alcoholic steatohepatitis; PBC, primary biliary cholangitis; PSC, primary sclerosing cholangitis; PSM, propensity score matched; OLT, orthotopic liver transplantation.

### 3.2. Propensity score matching

For the propensity score estimation, age at OLT, history of rejection ≤nine months after OLT, and liver disease etiology were chosen as covariates. The one-on-one matching led to 28 patients per group, nine patients in the intervention group (ESW) were lost since the caliper of 0.2 previously described was applied (20) (Figure 1). The SMD of all covariates was <0.1 except for age (SMD = 0.19), the variance ratio was also calculated for the numerical variables with a value <2, indicating a good covariate balance (Supplementary Figure S1). There were no statistical differences in the basal characteristics of the matched cohort. Patients with AIH, primary biliary cholangitis (PBC), and hepatocellular carcinoma (HCC), as well as those that had rejection before steroid discontinuation were excluded by the matching process (Table 1). In addition there were no differences regarding the MELD of the patients prior to the OLT, with a MELD median of 19.00 (11.75, 36.00) in the ESW compared to MELD median of 19.50 (8.00, 26.50) in the LSW (*p* = 0.22), and no difference on the incidence of biliary complications previous to the svLbx, 11/28 (39.3%) in the ESW compared to 9/28 (32.1%) in the LSW group (*p* = 0.78).

### 3.3. Immunosuppression

In the matched sample, patients with ESW discontinued steroids in a median of 9.4 (6.1, 12.6) months before the svLbx, on the other hand, those with LSW discontinued steroids in a median of 14.2 (1.3, 27.2) months after the svLbx and eight (28.6%) patients in this group discontinued steroids prior to the biopsy [median, 9.0 (4, 17)]**.** Patients with ESW had same levels of IS at the time of the svLbx, as measured by the aim IS simplified score (median, 4.0 (2.0, 4.0) vs. 4.0 (3.0, 5.0); *p* = 0.07). All patients in both groups had TAC as their primary IS. Most of the patients (27 (96.4%) in the ESW group were in dual therapy, whereas most of the patients (18 (64.3%) in the LSW group were on triple therapy at the time of the svLbx (Table 2).

**Table 2:** Immunosuppression, liver enzymes at svLbx, and DSA, before and after PSM analysis.

	Before PSM			After PSM		
	LSW after OLT (*n* = 72)	ESW after OLT (*n* = 37)	*p*-value	LSW after OLT (*n* = 28)	ESW after OLT (*n* = 28)	*p*-value
**Immunosuppression**						
Tacrolimus	71 (98.6)	37 (100.0)	1.00	28 (100.0)	28 (100.0)	1.00
Prednisolone	55 (76.4)	0 (0.0)	<0.001	20 (71.4)	0 (0.0)	0.001
Everolimus	8 (11.1)	7 (18.9)	0.41	5 (17.9)	6 (21.4)	1.00
Sirolimus	1 (1.4)	0 (0.0)	1.00	1 (3.6)	0 (0.0)	1.00
MMF	56 (77.8)	29 (78.4)	1.00	20 (71.4)	21 (75.0)	1.00
Monotherapy	0 (0.0)	1 (2.7)	<0.001	0 (0.0)	1 (3.6)	0.001
Dual therapy	25 (34.7)	36 (97.3)		10 (35.7)	27 (96.4)	
Triple therapy	47 (65.3)	0 (0.0)		18 (64.3)	0 (0.0)	
IS aim score at Biopsy	4.0 (3.5, 5.0)	4.0 (3.0, 4.0)	<0.001	4.0 (3.0, 5.0)	4.0 (2.0, 4.0)	0.07
Tacrolimus levels (ng/ml)	6.8 (5.3, 8.3)	5.7 (4.6, 7.5)	0.16	7.4 (5.4, 8.5)	5.6 (4.8, 7.7)	0.19
Everolimus levels (ng/ml)	4.8 (3.9,5.8)	4.1 (4.0,5.6)	0.82	5.0 (3.7, 5.5)	4.4 (4.0, 6.0)	0.86
Sirolimus levels (ng/ml)	16.8 (16.8, 16.8)	–	–	16.8 (16.8, 16.8)	–	–
MMF dose (mg/d)	1,000.0 (1000.0, 1000.0)	1,000.0 (1,000.0, 1000.0)	0.13	1,000.0 (937.0, 1000.0)	1,000.0 (750.0, 1000.0)	0.84
Prednisolone dose (mg/d)	5 (5.0, 5.0)	–	–	5 (2.5, 5.0)	–	–
AST (U/L)	26 (21.8, 31.6)	24 (20.0, 28.0)	0.14	23 (20.5, 34.0)	24 (19.5, 27.3)	0.31
ALT (U/L)	19 (14.2, 32.0)	17 (13.0, 22.0)	0.08	18 (14.8, 37.2)	17 (12.8, 22.2)	0.21
AP (U/L)	84 (64.0, 111.0)	90 (81.9, 105.5)	0.22	86 (69.0, 110.3)	89 (81.6, 101.5)	0.63
DSA^a^	10/25 (40.0)	4/17 (23.5)	0.44	3/7 (42.9)	1/13 (7.7)	0.20

Data is provided as *n* (%) or median [IQR].

ALT, alanine aminotransferase; AST, aspartate aminotransferase; AP, alkaline phosphatase; DSA, donor specific antibodies; ESW, early steroid withdrawal; IS, immunosuppression; LSW, late steroid withdrawal; MMF, mycophenolat-mofetil; PSM, propensity score matched; OLT, orthotopic liver transplantation.

^a^ DSA score was not available in all patients, the available number of patients with data for analysis is shown.

Five (17.9%) patients with ESW required reintroduction of the steroids during the follow-up, in a median of 27.0 (18.0, 82.5) months after discontinuation; one for clinical TCMR, two for other immunological diseases (vasculitis and IgA nephropathy), and two for side effects of the other IS drugs.

### 3.4. Histological outcomes

No differences in subclinical graft injury quantified by inflammation scores, i.e., RAI (median, 2.0 (1.0, 2.0) in the LSW compared to 1.0 (1.0, 1.0) in the ESW; *p* = 0.09) and Ishak modified HAI (median, 2.0 (1.0, 2.3) in the LSW compared to 1.50 (1.0, 2.0) in the ESW; *p* = 0.60) was detectable between the two groups (Table 3). The proportion of patients with subclinical TCMR was not different (*p* = 0.70) between patients in the ESW [*n* = 3 (10.7%)] and LSW (*n* = 5 (17.9%) (Table 4).

**Table 3:** Histological findings in liver graft surveillance biopsies.

		PSM		
		LSW after OLT (*n* = 28)	ESW after OLT (*n* = 28)	*p*-value
**Portal fields per biopsy**		11.0 (8.3, 15.0)	11.0 (10.0, 12.5)	0.75
Rejection activity index score (RAI)	Total RAI	2.0 (1.0, 2.0)	1.0 (1.0, 1.0)	0.09
	Portal	1.0 (1.0, 1.0)	1.0 (1.0, 1.0)	0.72
	Bile duct	0.0 (0.0, 1.0)	0.0 (0.0, 0.0)	0.05
	Venous	0.0 (0.0, 1.0)	0.0 (0.0, 0.0)	0.09
Ishak hepatitis activity Index (HAI)	Total Ishak HAI	2.0 (1.0, 2.3)	1.5 (1.0, 2.0)	0.60
	A (interface hepatitis)	0.0 (0.0, 1.0)	0.0 (0.0, 0.0)	0.06
	B (confluent necrosis)	0.0 (0.0, 0.0)	0.0 (0.0, 0.0)	0.32
	C (lobular inflammation)	0.5 (0.0, 1.0)	0.5 (0.0, 1.0)	0.90
	D (portal inflammation)	1.0 (0.8, 1.0)	1.0 (0.0, 1.0)	0.84
Liver allograft fibrosis Score (LAF)^a^	Total LAF	2.0 (1.0, 2.5) (*n* = 11)^a^	1.0 (0.0, 2.0) (*n* = 17)^a^	0.18
	Portal tract fibrosis	1.0 (1.0, 1.5)	1.0 (0.0, 1.0)	0.22
	Sinusoidal fibrosis	0.0 (0.0, 0.0)	0.0 (0.0, 0.0)	0.34
	Perivenular fibrosis	1.0 (0.0, 1.0)	0.0 (0.0, 0.0)	0.04
**Ishak fibrosis score**		1.0 (0.0, 1.0)	1.0 (0.0, 1.0)	0.57
**Fatty liver (>5%)**		2 (7.1)	7 (25.0)	0.15

Data is provided as no. (%) or median [IQR].

ESW, early steroid withdrawal; LSW, late steroid withdrawal; PSM, propensity score matched; OLT, orthotopic liver transplantation.

^a^ LAF score was not available in all patients, the available number of patients with data for analysis is shown.

**Table 4:** Histological diagnosis on surveillance biopsies.

	PSM		
	LSW after OLT (*n* = 28)	ESW after OLT (*n* = 28)	*p*-value
TCMR (RAI ≥ 1 + 1 + 1) *n* (%)	5 (17.9)	3 (10.7)	0.70
Significant fibrosis (Ishak F ≥ 2) *n* (%)	6 (21.4)	2 (7.1)	0.25
Re - cirrhosis Ishak (F ≥ 5) *n* (%)	0	0	
BANFFmini *n* (%)	7 (25)	9 (32.1)	0.77

Data is provided as no. (%) or median [range].

ESW, early steroid withdrawal; LSW, late steroid withdrawal; PSM, propensity score matched; OLT, orthotopic liver transplantation; TCMR, T-cell mediated rejection.

There was a non-significant trend for more fibrosis in the LSW group [Ishak ≥2, 6 (21.4%) compared to 2 (7.1%); *p* = 0.25]. Seven (25.0%) patients with LSW and nine (32.1%) patients with ESW fulfill the Banff criteria for IS reduction, without a significant statistical difference (Table 4).

In the matched cohort, one patient in each group developed a clinical TCMR (RAI = 4 plus elevated liver enzymes) after the svLbx during the period evaluated (eleven-36 months), both cases responded well to steroid therapy.

Not all patients in the LSW group were under continued steroid medication at the svLbx after PSM (Table 2). To exclude this bias, we repeated this analysis with a comparison of patients with and without steroid medication at the svLbx. The PSM resulted in two matched cohorts of 24 patients each (Supplementary data). Again, the steroid withdrawal was not associated with an increased graft injury (Supplementary Tables S3, S4). However, patients with continued steroid intake exhibited significantly but clinically not meaningful more interface hepatitis (median Ishak A: 0.0 (0.0, 1.0) in the group with steroids compared to 0.0 (0.0, 0.0) in the group without steroid; *p* = 0.01), lobular inflammation (median Ishak C: 1.0 (0.0, 1.0) in the group with steroid compared to 0.0 (0.0, 1.0) in the group without steroid; *p* = 0.03), and ductal inflammation (median RAI bile duct: 1.0 (0.0, 1.0) in the group with steroids compared to 0.0 (0.0, 0.0) in the group without steroids; *p* = 0.04), without differences in the total scores and in the final histological diagnosis (Supplementary Tables S3, S4).

### 3.5. Metabolic outcomes and survival

**Figure 2:**
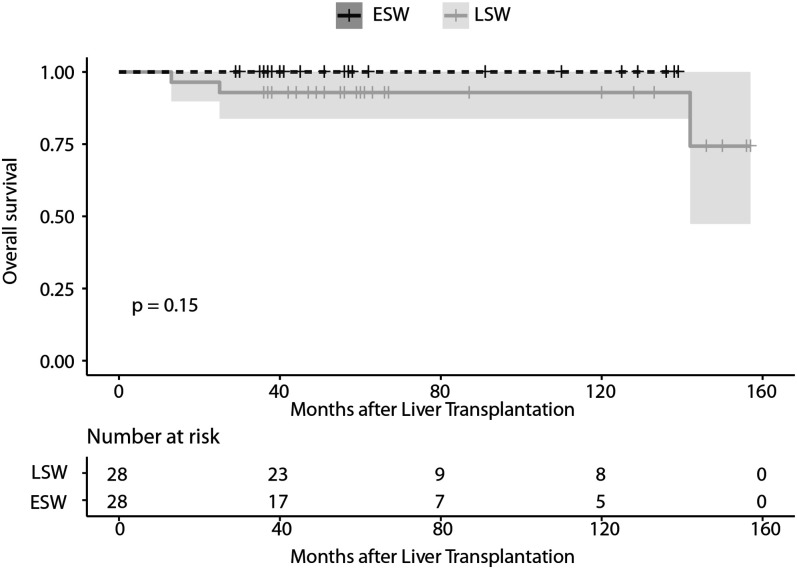
Survival curve of liver recipients in the PSM cohort in relation to the continuation or withdrawal of steroids at nine months after OLT. ESW, early steroid withdrawal; LSW, late steroid withdrawal; PSM, propensity score matched.

No differences in the frequency of NODAT [3 (10.7%) compared to 5 (17.9%); *p* = 0.70] and new-onset HTN (7 (25.0%) compared to 4 (14.3%); *p* = 0.50) at the time of the svLbx were observed between patient with LSW and ESW in the first year. There were also no differences in the glucose, cholesterol, creatinine, and ALT levels one year after OLT (Table 5). Regarding the overall survival, none of the patients with ESW died during the follow-up, whereas three of the patients with LSW died, two of them related to malignancy (*p* = 0.15), (Figure 2) this can be explained by the fact that the latter group had a longer total follow-up [median, 104.0 (58.0, 134.5) months compared to 40.0 (36.0, 58.0) months; *p* = 0.001)]. No graft losses were observed in either group during this follow-up.

**Table 5:** Metabolic outcomes at svLbx and blood values 1 year after OLT.

	PSM		
	LSW after OLT (*n* = 28)	ESW after OLT (*n* = 28)	*p*-value
NODAT	3 (10.7)	5 (17.9)	0.70
New-onset HTN post - OLT	7 (25.0)	4 (14.3)	0.50
Glucose (mg/dl) 1 year after OLT	90.0 (83.8, 106.0)	103.0 (93.5, 120.0)	0.11
Creatinine (mg/dl) 1 year after OLT	164.0 (98.8, 205.0)	166.0 (130.3, 202.0)	0.66
Cholesterol (mg/dl) 1 year after OLT	164.0 (98.8, 205.0)	166.0 (130.3, 202.0)	0.66
ALT 1 year after OLT	23.5 (16.8, 38.5)	18.5 (14.5, 26.8)	0.12

Data is provided as no. (%) or median [IQR].

ALT, alanine aminotransferase; CyA, cyclosporin A; ESW, early steroid withdrawal; HTN, hypertension; LSW, late steroid withdrawal; NODAT, new onset diabetes after transplantation, PSM, propensity score matched; OLT, orthotopic liver transplantation.

## 4. Discussion

Although subclinical immune-mediated graft injury has been previously neglected in OLT, there is a growing body of evidence suggesting that these histopathological changes could have greater relevance in long-term graft outcomes than previously thought (9, 10). Despite the long history of steroid use in transplantation, there is no previous attempt to assess the risk of subclinical graft injuries following steroid withdrawal in OLT within svLbx.

To assess this question, we retrospectively evaluated a prospectively collected cohort of LR with representative svLbx between eleven and 36 months after OLT with a PSM analysis to balance the differences between patients who withdrew steroids in the first nine months after OLT vs. those that did not. The main finding of this study is that steroid withdrawal has no obvious negative effect on the prevalence of subclinical graft injury in svLbx.

Previous trials of steroid-free or short-term steroid therapies in OLT, seldom performed svLbx and their primary outcome was the development of clinical rejection, rather than the evaluation of subclinical damage (5–7). Pageaux et al. compared CyA monotherapy vs. CyA plus steroids and performed protocol biopsies twelve months after OLT, reporting similar histological findings in the surveillance biopsies, however, the histopathology reports were quantitative and did not present detailed characterization (5). In addition, CyA is associated with lower survival rates after OLT compared to TAC and thus its use is usually restricted to TAC intolerance or patients with a PBC (23). Belli et al. and Ramirez et al. also performed prospective trials of free steroid therapies with svLbx, however, the reports of the histological findings in both papers were limited and the cohorts were either dominated by HCV with reinfections or CyA was the primary CNI (6, 7). In this report, we decided to exclude patients with CyA in order to have a more representative cohort of the current IS standard, usually with TAC as the primary IS. In addition, the use of CyA was associated with more graft injury in the preliminary analysis of our cohort (data not shown), which could have been an additional source of bias. After the PSM, IS in both groups was virtually the same, not only on the type of medication but also in their trough concentrations, except for the presence of steroids; avoiding confounding factors associated with the concomitant IS. However, it is worth noting that approximately one-third of patients in the LSW were not taking steroids at the time of biopsy, meaning that they had discontinued steroids between the nine-month cut-off and svLbx. Although this resembles the real world, it might also introduce further bias. To further reduce the possibility of bias, we performed a secondary analysis comparing patients with and without steroids at the time of biopsy. This more stringent patient selection resulted in a smaller cohort but confirmed the finding that svLbx without steroid medication did not have an increased graft injury pattern within the first 3 years after OLT. Unexpectedly, patients on steroids had more inflammation in some items of the mHAI score and the RAI score, with no difference in overall scores. We can only speculate on the reasons for these differences because, apart from the presence of steroids, the other covariates were the same between the groups. However, this does not detract from the fact that steroid withdrawal is not a risk factor for subclinical injury.

In general, inflammation was low in both groups, and up to one-third of patients in both groups met the BANFFmini criteria for IS reduction. This means that, even in patients with previous steroid discontinuation, the possibility of continuing to safely minimize IS is comparable to that in patients with long-term steroid treatment. Thus, in this group of patients without an autoimmune background, long-term steroid therapy seems to not necessary. We also did not find any difference regarding the risk of developing fibrosis, this was however expected due to the relative early time point of the evaluation after OLT.

Unlike previous evidence that has shown a reduction in NODAT and new-onset HTN in free steroid or short-term steroid protocols (8), our study did not show this benefit, this may be because the discontinuation of steroids was not as early as other protocols that showed these benefits (8). The likelihood of preventing metabolic complications is probably higher when steroids are suspended early after OLT. It is important to note, that the lack of steroids was not compensated by more aggressive IS using other agents, which may also increase the risk of metabolic diseases and chronic kidney damage.

Our retrospective analysis of real life data has some obvious limitations. First, although it is a prospectively collected cohort, the analysis of steroid discontinuation was done retrospectively, increasing the risk of selection bias inherent to real-world studies without a harmonized IS regime. In addition, even though the histology and the IS are well characterized, there was no systematic documentation of infection events, which are important factors in IS management, in our database. Furthermore, the biopsies were not taken at the same exact point, 17 (12.8, 25.0) months after OLT in the case of LSW and 13 (12.0, 17.0) months after OLT in the ESW, however, it is important to mention that the time point of the biopsies was no cofounded by previous clinical events (e.g., lack of compliance, previous rejection events, etc), we did not include any indication biopsy. We tried to compensate for these factors by balancing the groups to compare through the use of PSM. Also, the time-point of the biopsy after OLT was better balanced after the PSM, 15.0 (12.0, 24.3) months after OLT in the case of LSW and 13.0 (12.0, 17.3) months after OLT in the ESW. Additionally, we only included biopsies within a 24-month time window (between 11 and 36 months after OLT). We recently demonstrated that there were no significant odds of relevant graft injury such as subclinical TCMR, fibrosis, or absence of relevant graft injury (BANFFmini) within the first 3 years (10).

We included early acute TCMR and the underlying liver disease in the PSM as cofounders for a continued steroid application. So, patients with AIH, PBC, and a history of acute TCMR were excluded during the matching process. It is important to emphasize that the current analysis is not valid for these groups, with a higher risk of immunogenic reaction, i.e., AIH and PBC. Likewise PSC is a bit underrepresented in our PSM cohort. Currently, steroids were continued until the first svLbx around year one after OLT in patients with AIH and early acute TCMRs at our center. Steroids are discontinued when there is no relevant graft injury. It is relevant to mention, however, that in a previous report, we were able to demonstrate that individualized adjustment of IS by svLbx is also safe in autoimmune patients (11).

Another limitation is the limited number of patients, which restricts the power of the study, and the possibility of including more covariates in the PSM. However, it is unlikely that larger svLbx cohorts will be available in the near future including routine steroid continuation to do this type of analysis. Finally, no reliable data regarding the quality of life was collected, and therefore reported, which are important variables when discussing any IS management. This might be of importance, because steroid use is associated with reduced QoL in AIH patients (24).

Lastly, we show a high survival in both groups, only three patients in the LSW died during the follow-up. This could be the result of a selection bias of the more compliant patients who participated in the voluntary surveillance biopsy program. This higher survival has been observed in previous surveillance biopsy cohorts from other centers (25). However, since both groups are part of the same surveillance program, this selection bias should not represent a limitation of the study for the comparison between the groups.

In conclusion, steroid withdrawal within the first nine months post-transplantation, as assessed by surveillance biopsies, is a safe maneuver and does not increase the risk of subclinical graft injury, significant fibrosis, and graft hepatitis in OLT.

## Data Availability

The raw data supporting the conclusions of this article will be made available by the authors, without undue reservation.
