# Supplementary material for: Impact of steroid withdrawal on subclinical graft injury after liver transplantation: A propensity score-matched cohort analysis
Source: Front Transplant. 2023 Feb 22;2:1124551. doi: 10.3389/frtra.2023.1124551 (PMC11235343; doi:10.3389/frtra.2023.1124551)
Supplement: Supplementary file 1 [file Table1.docx]

**Impact of steroid withdrawal on subclinical graft injury after liver transplantation: A propensity score-matched cohort analysis**

Alejandro Campos-Murguia1, Emily A Bosselmann1, Björn Hartleben2, Heiner Wedemeyer1, Bastian Engel1, *Richard Taubert1, *Elmar Jaeckel1, #

**Affiliations:**

^1^ Department of Gastroenterology, Hepatology, and Endocrinology, Hannover Medical School, Hannover, Germany

^2^ Institute for Pathology, Hannover Medical School, Hannover, Germany

# Current address: Ajmera Transplant Center, Toronto General Hospital, United Health Network, University of Toronto, Canada.

* Equal senior authors

**Impact of steroid presence or absence as a risk factor for subclinical graft injuries.**

Secondary analysis comparing patients with and without steroids at the moment of the surveillance biopsy, patients who suspended steroids within the 3 months before the biopsy where excluded, to assure a stable immunologic milieu.

Data showing original cohort and PSM using Age, etiology and rejection before the svLbx as covariates for the propensity score.

**Supplementary Table 1**. General characteristics of the groups before PSM and after PSM.

|  |  |  |  |  |  |  |  |  |
| --- | --- | --- | --- | --- | --- | --- | --- | --- |
|  | Before PSM | | |  | | After PSM | | |
|  | Steroid at biopsy | Steroid free at biopsy | p value |  | | Steroid at biopsy | Steroid free at biopsy | p value |
|  | (n = 55) | (n = 49) |  |  | | (n = 24) | (n = 24) |  |
| Male Sex | 36 (65.5) | 26 (53.1) | 0.28 |  | | 17 (70.8) | 13 (54.2) | 0.37 |
| Age at LT (years) | 48.0 [17.0, 65.0] | 52.0 [28.00, 67.0] | **0.03** |  | | 51.5 [46.0, 59.5] | 52.5 [48.5, 57.3] | 0.95 |
| Etiology |  |  | **0.03** |  | |  |  | 0.70 |
| AIH | 8 (14.5) | 0 (0.0) |  |  | | 0 (0.0) | 0 (0.0) |  |
| Alcohol | 6 (10.9) | 7 (14.3) |  |  | | 1 (4.2) | 3 (12.5) |  |
| Cryptogenic | 6 (10.9) | 7 (14.3) |  |  | | 4 (16.7) | 4 (16.7) |  |
| Cystic disease | 6 (10.9) | 5 (10.2) |  |  | | 6 (25.0) | 4 (16.7) |  |
| HBV | 5 (9.1) | 8 (16.3) |  |  | | 4 (16.7) | 5 (20.8) |  |
| HCC | 0 (0.0) | 1 (2.0) |  |  | | 0 (0.0) | 0 (0.0) |  |
| HCV | 5 (9.1) | 6 (12.2) |  |  | | 4 (16.7) | 1 (4.2) |  |
| Metabolic disease | 4 (7.3) | 4 (8.2) |  |  | | 2 (8.3) | 3 (12.5) |  |
| NAFLD/NASH | 1 (1.8) | 4 (8.2) |  |  | | 1 (4.2) | 0 (0.0) |  |
| Other | 2 (3.6) | 3 (6.1) |  |  | | 1 (4.2) | 3 (12.5) |  |
| PBC | 1 (1.8) | 3 (6.1) |  |  | | 0 (0.0) | 0 (0.0) |  |
| PSC | 11 (20.0) | 1 (2.0) |  |  | | 1 (4.2) | 1 (4.2) |  |
| BMI at OLT (kg/m^2^) | 23.8 [14.6, 45.0] | 23.36 [17.5, 34.0] | 0.51 |  | | 24.9 [22.1, 27.4] | 21.7 [20.1, 26.4] | 0.10 |
| DM pre-OLT | 7 (12.7) | 5 (10.2) | 0.93 |  | | 5 (20.8) | 1 (4.2) | 0.19 |
| HAT pre-OLT | 15 (27.3) | 15 (30.6) | 0.87 |  | | 10 (41.7) | 8 (33.3) | 0.77 |
| Follow-up Time | 95.0 [13.0, 158.0] | 45.00 [29.0, 141.0] | **<0.001** |  | | 63.0 [47.3, 126.5] | 45.5 [37.0, 73.0] | 0.13 |
| Rejection before biopsy | 19 (34.5) | 3 (6.1) | **0.001** |  | | 0 (0.0) | 2 (8.3) | 0.47 |
|  |  |  |  |  | |  |  |  |
| Months from OLT to surveillance biopsy | 15.0 [12.0, 24.0] | 16.0 [12.0, 24.0] | 0.714 |  | | 14.0 [12.0, 20.0] | 17.5 [12.8, 24.3] | 0.09 |

*Note:* Data is provided as no. (%) or median [IQR].

Abbreviations: AIH, autoimmune hepatitis; BMI, body max index; DM, diabetes mellitus; ESW, early steroid withdrawal; HBV, hepatitis B virus; HCC, hepatocellular carcinoma; HCV, hepatitis C virus; HTN, hypertension; LSW, late steroid withdrawal; NAFLD, non-alcoholic liver disease; NASH, non-alcoholic steatohepatitis; PBC, primary biliary cholangitis; PSC, primary sclerosing cholangitis; PSM, propensity score matched; OLT, orthotopic liver transplantation.

**Supplementary Table 2**. Immunosuppression characteristics, liver enzymes at svLbx, and DSA

of the groups before PSM.

|  | Before PSM | | |  | After PSM | | |
| --- | --- | --- | --- | --- | --- | --- | --- |
|  | Steroid at biopsy (n = 55) | steroid free at biopsy (n =49) | p value |  | Steroid at biopsy (n = 24) | steroid free at biopsy (n =24) | p value |
| Immunosuppression |  |  |  |  |  |  |  |
| Tacrolimus | 54 (98.2) | 49 (100.0) | 1.00 |  | 24 (100.0) | 24 (100.0) | 1.00 |
| Prednisolone | 55 (100.0) | 0 (0.0) | **<0.001** |  | 24 (100.0) | 0 (0.0) | **<0.001** |
| Everolimus | 7 (12.7) | 8 (16.3) | 0.81 |  | 4 (16.7) | 3 (12.5) | 1.00 |
| Sirolimus | 1 (1.8) | 0 (0.0) | 1.00 |  | 1 (4.2) | 0 (0.0) | 1.00 |
| MMF | 40 (72.7) | 40 (81.6) | 0.40 |  | 16 (66.7) | 21 (87.5) | 0.17 |
| Monotherapy | 0 (0.0) | 1 (2.0) | **<0.001** |  | 0 (0.0) | 0 (0.0) | **<0.001** |
| Dual therapy | 8 (14.5) | 48 (98.0) |  |  | 3 (12.5) | 24 (100) |  |
| Triple therapy | 47 (85.5) | 0 (0.0) |  |  | 21 (87.5) | 0 (0.0) |  |
| IS aim score at biopsy | 5.0 [3.8, 5.0] | 4.0 [3.0, 4.0] | **<0.001** |  | 4.8 [3.6, 5.0] | 3.8 [3.0, 4.0] | **0.004** |
| Tacrolimus levels | 6.8 [5.0, 8.4] | 6.0 [5.1, 7.4] | 0.28 |  | 7.3 [4.8, 8.7] | 5.7 [5.2, 7.1] | 0.22 |
| Everolimus levels | 4.6 [3.9, 5.3] | 4.4 [4.0, 6.5] | 0.56 |  | 5.3 [4.7, 5.8] | 4.1 [3.9, 5.3] | 0.59 |
| Sirolimus levels | 16.8 [16.8, 16.8] | - | - |  | 16.8 [16.8, 16.8] | - | - |
| Prednisolone dose | 5.0 [5.0, 5.0] | - | - |  | 5.0 [2.9, 5.0] | - | - |
| MMF dose | 1000.0 [1000.0, 1500.0] | 1000.0 [750.0, 1000.0] | **0.01** |  | 1000.0 [1000.0, 1125.0] | 1000.0 [500.0, 1000.0] | 0.08 |
| AST (U/L) | 26.0 [22.0, 32.3] | 22.8 [18.0, 28.0] | **0.01** |  | 26.5 [19.8, 33.8] | 22.4 [19.5, 26.2] | 0.15 |
| ALT (U/L) | 20.8 [16.1, 36.3] | 16.0 [12.0, 22.0] | **0.01** |  | 18.5 [15.89, 37.1] | 15.5 [12.3, 20.5] | **0.04** |
| AP (U/L) | 84.4 [63.2, 117.4] | 90.0 [80.8, 104.2] | 0.43 |  | 79.3 [60.31, 102.8] | 90.0 [77.6, 100.0] | 0.18 |
| DSA* | 6/17 (35.3) | 7/22 (31.8) | 1.00 |  | 4/8 (50.0) | 2/8 (25.0) | 0.61 |

*Note:* Data is provided as no. (%) or median [IQR]. Abbreviations: ALT, alanine aminotransferase; AST, aspartate aminotransferase; AP, alkaline phosphatase; DSA, donor specific antibodies; IS, immunosuppression; MMF, Mycophenolat-Mofetil; PSM, propensity score matched. * DSA score was not available in all patients, the available number of patients with data for analysis is shown.

**Supplementary Table 3.** Histological findings in liver graft surveillance biopsies.

|  | |  |  | PSM |  |
| --- | --- | --- | --- | --- | --- |
|  | |  | Steroid at biopsy  (n = 24) | steroid free at biopsy (n = 24) | p-value |
| Portal fields per biopsy | | |  |  |  |
| Rejection activity index score (RAI) | Total RAI | | 1.5 [1.0, 2.0] | 1.0 [1.0, 1.3] | 0.41 |
|  | Portal | | 1.0 [1.0, 1.0] | 1.0 [1.0, 1.0] | 0.28 |
|  | Bile duct | | 1.0 [0.0, 1.0] | 0.0 [0.0, 0.0] | **0.04** |
|  | Venous | | 0.0 [0.0, 0.8] | 0.0 [0.0, 0.0] | 0.28 |
| Ishak  hepatitis  activity  Index (HAI) | Total Ishak HAI | | 2.0 [1.0, 3.0] | 2.0 [1.0, 2.0] | 0.16 |
|  | A (interface hepatitis) | | 0.0 [0.0, 1.0] | 0.0 [0.0, 0.0] | **0.01** |
|  | B (confluent necrosis) | | 0.0 [0.0, 0.0] | 0.0 [0.0, 0.0] | 0.32 |
|  | C (lobular inflammation) | | 1.0 [0.0, 1.0] | 0.0 [0.0, 1.0] | **0.03** |
|  | D (portal inflammation) | | 1.0 [1.0, 1.0] | 1.0 [1.0, 1.0] | 0.69 |
| Liver  allograft  fibrosis  Score (LAF)* | Total LAF | | 1.5 [0.3, 2.0] (n=10) | 1.0 [0.3, 2.0] (n=14) | 0.74 |
|  | Portal tract fibrosis | | 1.0 [0.3, 1.0] | 1.0 [0.3, 1.0] | 1.00 |
|  | Sinusoidal fibrosis | | 0.0 [0.0, 0.0] | 0.0 [0.0, 0.0] | 0.36 |
|  | Perivenular fibrosis | | 0.0 [0.0, 1.0] | 0.0 [0.0, 1.0] | 0.94 |
| Ishak fibrosis score | | | 0.5 [0.0, 1.0] | 0.0 [0.0, 1.0] | 0.31 |
| Fatty liver (> 5%) | | | 1 (4.2) | 2 (8.3) | 1.00 |

*Note:* Data is provided as no. (%) or median [IQR].

Abbreviations: PSM, propensity score matched; OLT.

* LAF score was not available in all patients, the available number of patients with data for analysis is shown

**Supplementary Table 4.** Histological diagnosis on surveillance biopsies.

|  |  |  | PSM |  |
| --- | --- | --- | --- | --- |
|  |  | Steroid at biopsy  (n = 24) | steroid free at biopsy (n = 24) | p-value |
| TCMR (RAI ≥ 1+1+1) |  | 5 (20.8) | 3 (12.5) | 0.70 |
| Significant fibrosis (Ishak F ≥ 2) |  | 4 (16.7) | 1 (4.2) | 0.35 |
| BANFFmini |  | 8 (33.3) | 8 (33.3) | 1.00 |

*Note:* Data is provided as no. (%) or median [IQR].

Abbreviations: PSM, propensity score matched; TCMR, T-cell mediated rejection.

**Suppl. Figure 2. Data of the PSM, Etiology, Age and Rejection before the svLbx as covariates.**

A. Distribution of matched and unmatched subjects of each group relative to their propensity score. B. Proportion of the sample before and after the PSM relative to the propensity score. C. Data of the covariate balance, each point represents the SMD of the corresponding covariate before and after the PSM. A SMD < 0.1 represents a proper balance. Abbreviations: ESW, early steroid withdrawal; HBV, hepatitis B virus; HCC, hepatocellular carcinoma; HCV, hepatitis C virus; LSW, late steroid withdrawal; NAFLD, non-alcoholic liver disease; NASH, non-alcoholic steatohepatitis; PBC, primary biliary cholangitis; PSC, primary sclerosing cholangitis; PSM, propensity score matched; OLT, orthotopic liver transplantation.

**
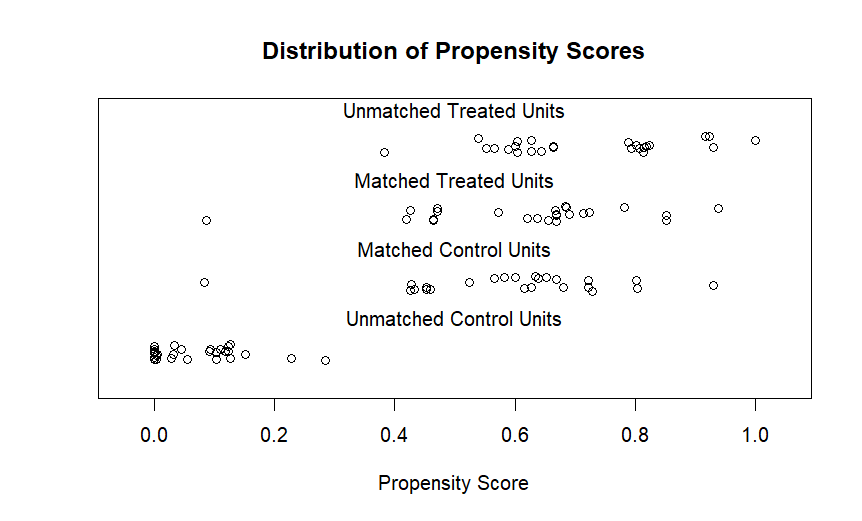
A)**

**B)**

**
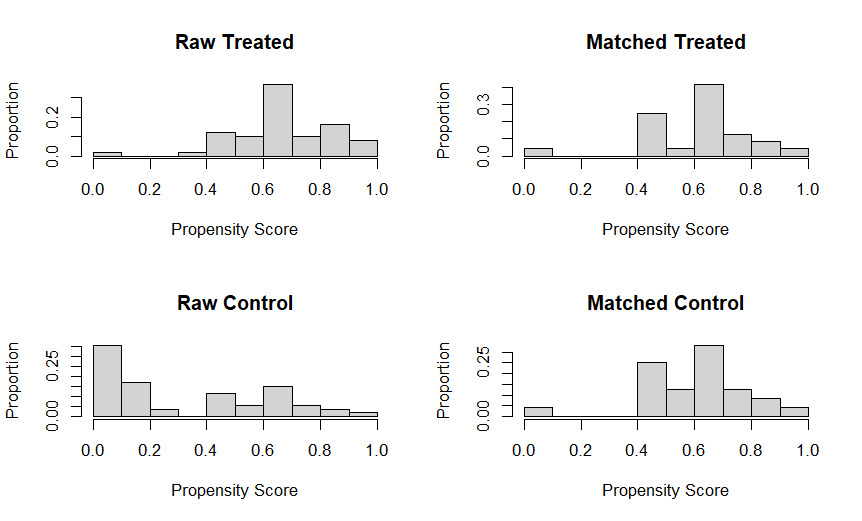
**

**
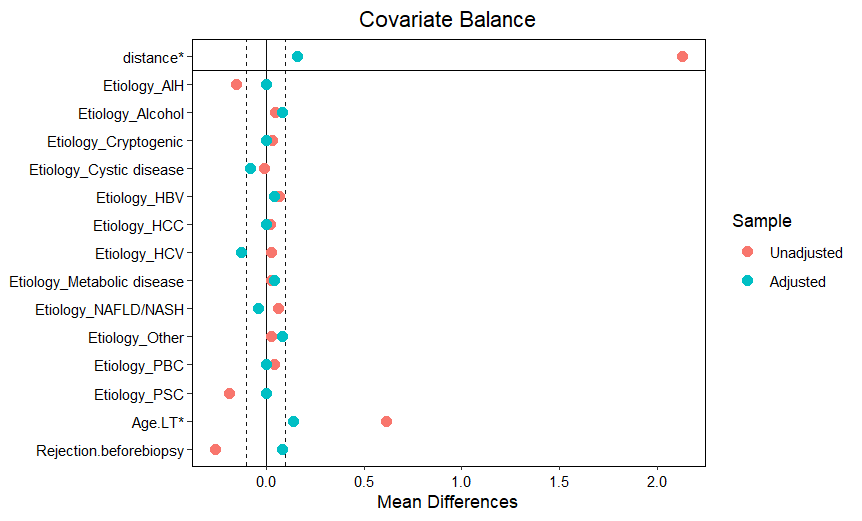
C)**
