# Supplementary figures and images for: Impact of steroid withdrawal on subclinical graft injury after liver transplantation: A propensity score-matched cohort analysis
Source: Front Transplant. 2023 Feb 22;2:1124551. doi: 10.3389/frtra.2023.1124551 (PMC11235343; doi:10.3389/frtra.2023.1124551)

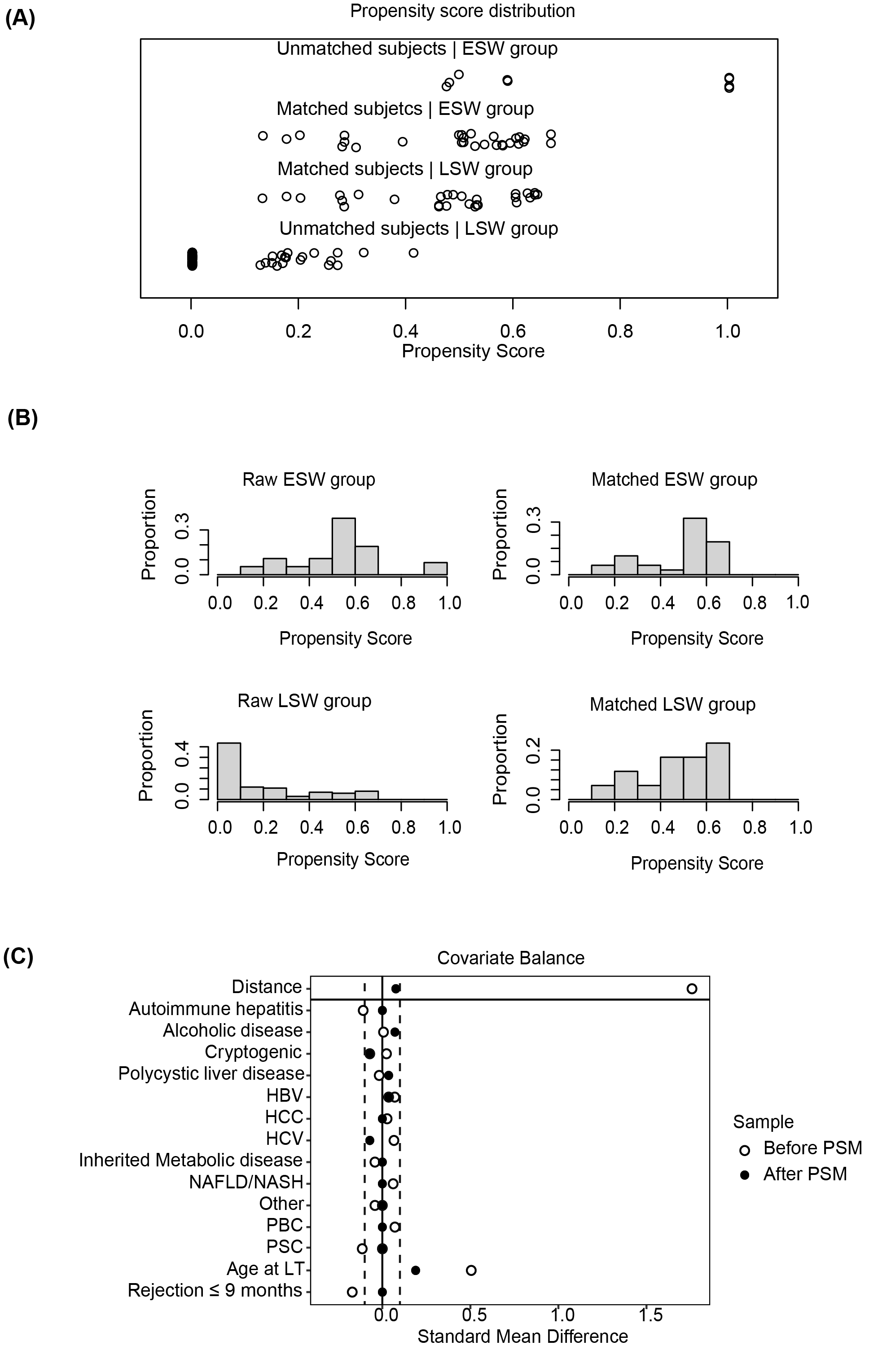

Supplement: Supplementary file 2 [file Image1.tif]
